# The Ant Genus *Oxyopomyrmex* Wheeler (Formicidae, Myrmicinae) from the Peninsula Iberica: Two New Species and New Distributional Data †

**DOI:** 10.3390/insects16060581

**Published:** 2025-05-30

**Authors:** Joaquín L. Reyes-López

**Affiliations:** Department of Botany, Ecology and Plant Physiology, University of Córdoba, Campus of Rabanales, Building C-4, 14071 Córdoba, Spain; cc0reloj@uco.es

**Keywords:** *Oxyopomyrmex*, Myrmicinae, revision, Iberian Peninsula

## Abstract

Two new species of the genus *Oxyopomyrmex* are described from Spain (Iberian Peninsula). The first belongs to the magnus group—characterized by a head more wide than long—and is distinguished by strongly developed cephalic striations, pale coloration (light brown) and a relatively small body size. To date, it has been recorded in the provinces of Jaén and Granada. The second species is even smaller in size, with very faint cephalic striations and short, triangular propodeal spines—morphological features that clearly set it apart from all previously known species. Its current distribution appears to be restricted to the province of Huelva, near the Atlantic coast, including the iconic Doñana National Park. With these additions, the number of *Oxyopomyrmex* species known from Spain increases from two to four. To date, sampling efforts have only focused on the southern part of the country, suggesting that additional, undiscovered species may still exist.

## Simple Summary

Some ant species remain poorly studied due to characteristics such as low population density and cryptic behavior. This is the case for the genus *Oxyopomyrmex* in the Iberian Peninsula. Through a detailed biometric analysis incorporating scanning electron microscopy (SEM) and novel morphological variables, we were able to describe two new species. One of the key advantages of SEM is its ability to produce images with exceptional depth of field, surpassing that of conventional optical microscopy.

## 1. Introduction

The genus *Oxyopomyrmex* André, 1881, is made up of species of small myrmicinae ants (currently placed in the tribe Stenammini) found in the arid areas of the Mediterranean region. Furthermore, it is a genus about which there is very little, fragmentary information, a consequence of its low density. This genus was first established upon the discovery of *O. oculatus* André, 1881, based on a single worker collected in Israel [1]. Subsequently, *O. saulcyi* Emery, 1989, was described in Banyuls, France (eastern Pyrenees) [2]. Between 1904 and 1936—a span of approximately thirty years—nine further species were recorded, six of which resulted from the work of Félix Santschi. It was not until 2015—nearly eighty years later—that five additional species were added, bringing the total number of currently recognized valid species to sixteen [3]. No further species have been described in the past ten years.

The morphology of this genus allows it to be easily distinguished from other Mediterranean genera because it has (1) characteristic large oval compound eyes, pointed anteroventrally, with an anterior margin near the mandibular insertions and (2) antennae with 11 segments.

It is a genus closely related phylogenetically to *Goniomma* Emery, 1895 [4], from which it is mainly distinguished by the number of antennal segments (11 in *Oxyopomyrmex*, 12 in *Goniomma*). Their diet is similar: they all feed mainly on seeds and forage individually. Their distribution in the Mediterranean arc is also very similar [5,6].

Species of the genus *Oxyopomyrmex* André, 1881, prefer open, herbaceous and arid environments with sparse vegetation [5]. Nests are located on the ground (usually sand or clay), sometimes under stones. In most species, the nest entrance is surrounded by a small crater composed of plant remains and soil [5,6].

In Spain, before the last revision [1], the presence of two species was recorded: *Oxyopomyrmex santschii* Forel, 1904, and *Oxyopomyrmex saulcyi* Emery, 1889. After the revision, *Oxyopomyrmex magnus* Salata & Borowiec, 2015, was described, and *O. santschii* was considered a junior synonym of the current valid taxon *O. saulcyi* [3].

To better understand the myrmecofauna of Spain, as well as to deepen knowledge of its diversity and geographic distribution, a field survey was conducted focusing on nests of the genus *Oxyopomyrmex* at various locations in the southern part of the country. This region, characterized by arid and semi-arid climatic conditions, represents a potentially favorable habitat for species of this genus, whose biology and ecology remain poorly understood. The sampling included a range of open habitats, primarily with sparse herbaceous vegetation, and was carried out across several provinces, with particular emphasis on areas that had been poorly explored in previous studies. The primary objective was to identify potential new species or morphologically distinct populations that could contribute valuable information on the species richness of this group in Spain.

## 2. Materials and Methods

**Identification of samples**

Workers were identified following the detailed keys published in [3]. In addition, images from AntWeb (https://www.antweb.org/, accessed on 1 March 2025) were used as references, as well as specimens deposited in various collections. Other additional information comes from [7].

Photos were taken using a JSM 6300 Scanning Electron Microscope (JEOL Ltd., Tokyo, Japan) (morphometric measurements as well). SEM microscopes provide a depth of field that cannot be matched by conventional optical microscopy. This microscope model allows the movement of the sample observation platform in the spatial axes (X, Y, Z). Between 70 and 500 magnifications were used. This microscope belongs to the central research support services (SCAI) of the University of Córdoba. Additional observation and photography of the specimens were performed with the Leica S6D stereomicroscope (Leica Microsystems, Wetzlar, Germany) (×80). The photographs taken by Paco Alarcón required focus stacking (see the details of his technique at https://pacoalarcon-hormigas.blogspot.com/, accessed on 1 March 2025).

To quantify some of the variables, the first step was to obtain a photograph. Subsequently, specific software, such as ImageJ 1.53e [8], had to be used.

**Measurements** (sensu [3])

Measurements marked with a (*) are new, and I believe that they have never been used before.

HL—head length: measured in full-face view in a straight line from the mid-point of the anterior clypeal margin to the mid-point of the posterior margin.

**Figure 1:**
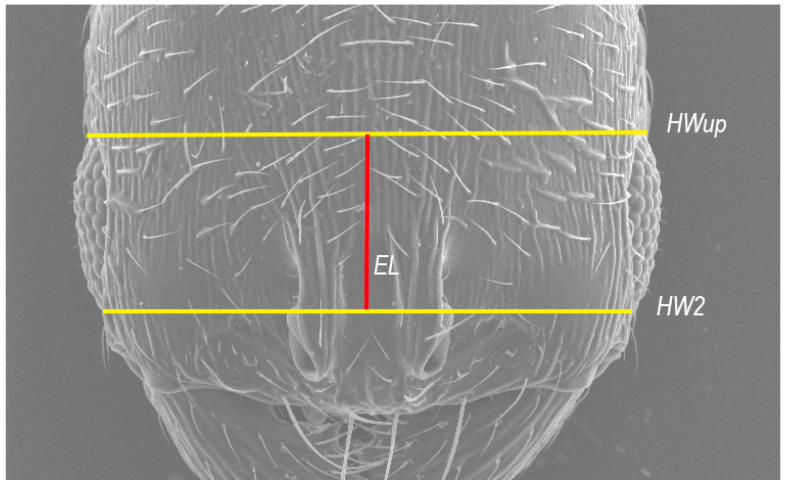
SEM image showing the position of the HWup, HW2 and EL variables for *O. arenarius*. Created by J.L. Reyes.

HWup—upper head width: measured in full-face view in a straight line directly above the eyes (Figure 1).

HW2—head width: measured in full-face view directly under the eyes.

EL (*)—average between the maximum visible eye length, in full-face view. Graphically, EL represents the straight line connecting the centers of the lines corresponding to the HWup and HW measurements (see Figure 1 for a better understanding).

SL—scape length: maximum straight-line length of the scape.

ML—mesosoma length: measured as diagonal length from the anterior end of the neck shield to the posterior margin of the propodeal lobe.

MH (*)—metanotal height: measured from the upper edge of the metanotal groove to the upper point of the mesopleural–coxal excavation in lateral view.

**Figure 2:**
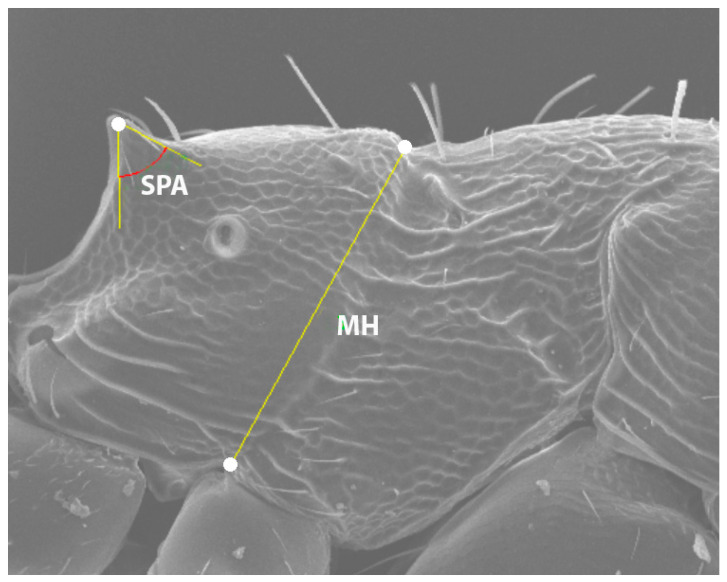
SEM image showing the position of the SPA and MH variables for *O. arenarius*. Created by J.L. Reyes.

SPA (*)—spine angle: Maximum angle included within the spine (lateral view), with the vertex at the apex of the spine. The sides of the angle progressively diverge until each intersects the lateral contour of the spine at a single point, forming a tangent line (see Figure 2).

SDL—spiracle-to-declivity length: minimum distance from the center of the propodeal spiracle to the propodeal declivity in lateral view.

PSL—propodeal spine length: measured from the center of the propodeal spiracle to the tip of the propodeal spine in lateral view.

PH—petiole height: maximum height of the petiole in lateral view.

PL—petiole length: measured from the spiracle to the most distal point in lateral view.

**Indexes**

HI—cephalic index: HWup/HL × 100.

SI—scape index: SL/HL × 100.

SPI—propodeal spine index: PSL/SDL × 100.

The format used for the measurements is µm: 178 ± 15 (163–211) = the average measurement ± standard error (min.–max.).

**Examined specimens are housed in the following collections:**

MNCN—Madrid Natural Science Museum. Spain.

CCZ-UGR—University of Granada. Spain.

RLC—Reyes-López collection, University of Córdoba. Spain.

**Statistical analysis**

For the biometric study, four taxonomic groups were used. The two species of this genus considered valid for Spain were used, and the other two groups were used to constitute potential new species (Table 1).

The nests were classified into these four groups based on an initial visual inspection. The differences between them were so evident that no prior exploratory statistical analysis was required to determine the possible number of groups. Therefore, the analyses were conducted solely for confirmatory purposes, which simplifies the process.

A linear discriminant analysis (LDA) was used based on morphometric variables and using the species as classifier variables. Each row of the database represents the measurements of one worker. Discriminant analysis is used for dimensionality reduction, as it identifies the most important variables for distinguishing between groups (species).

LDA was conducted in R (ver. 4.1.1) (R Core Team (2021)) using the “lda” function of the package MASS (version 7.3–64) [8]. It is based on finding the linear combinations of features that best separate the classes in the data set. All variables may not be equally important in separating taxonomic groups. To this end, a stepwise selection of variables can be performed using Wilk’s lambda criterion [9]. The “greedy.wilks” function, package klaR (version 1.7-3), was used. The initial model is defined by starting with the variable that most separates the groups. The model is subsequently expanded to include more variables according to Wilk’s lambda criterion; the one that minimizes the Wilk’s lambda of the model that includes the variable is selected if its *p*-value is still statistically significant.

The results of the LDA were plotted using the “ggplot2” package (version 3.5.0) and the “geom_polygon” function, to draw a convex hull polygon around the points (=workers) of each group.

**Collinearity and its impact**

In morphological studies, multicollinearity refers to a situation where two or more morphological variables are highly correlated with each other [10]. Multicollinearity can inflate standard errors and make it difficult to interpret the effects of individual variables. Stepwise discriminant analysis may sometimes eliminate collinear variables, but its primary objective is to identify the most informative subset of predictors. The level of multicollinearity in the data can be assessed using various statistical techniques, such as the determinant of the correlation matrix among variables or the Variance Inflation Factor (VIF). When the determinant approaches zero or the VIF exceeds 10, the degree of multicollinearity is considered problematic [10,11,12].

## 3. Results

**Biometric analysis**

Biometric comparisons were made with a sample of 15 nests (Table 1): 4 nests of *Oxyopomyrmex saulcyi*, 4 nests of *Oxyopomyrmex magnus* and 7 nests of the new species. Therefore, comparisons were made with the species present in Spain. Between four and five workers were measured in each nest. The total sample was 57 workers.

**Figure 3:**
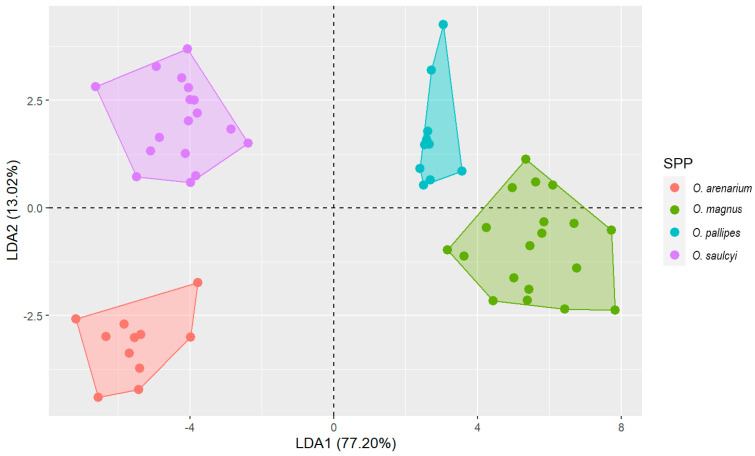
Results of the stepwise linear multivariate discriminant analysis. Each point in the plot corresponds to a specimen measured. Convex hull polygon refers to the lines drawn to form a bounding box around the outermost points in each group.

The LDA showed a significant result (Wilks’ lambda: 0.005, F (15,135) = 53.052, *p* < 0.0001), using only five morphological variables: HI, EL, SPA, SPI and PH (order resulting from the step-by-step analysis). The first eigenvalue accounted for 77% of the variance. All pairwise comparisons across all four species were significant (*p* < 0.001). Finally, 100% of the original grouped cases were correctly classified (see Figure 3). One of the variables with the greatest weight in the LDA was SPA (internal angle of the propodeal spines). This highlights the value of using this variable in future revisions.

The value of the determinant (D) was nearly zero, and the VIF values were below 10 for only three variables, indicating the presence of multicollinearity. After applying stepwise LDA, the determinant increased to 0.47, and the VIF values ranged from 1.50 to 2.08, well below the threshold of 10.

**Description of the species**

***Oxyopomyrmex arenarius* sp. nov.**

**Etymology**. The term “arenarius” derives from the Latin {arenarius, -a, -um}, meaning “of sand” or “sandy”. So far, all nests have been detected in sandy soils (dune origin).

**Type locality**. Doñana Biological Station (EBD-CSIC). Spain.

**Type material**. Holotype worker (pin): SPAIN|Doñana|18-XI-2017|RLC; two paratype workers: the same data as holotype (MNCN). The rest of the paratypes are in the author’s collection.

**Measurements and ratios** (in microns, *n* = 11): HL: 595.5 ± 9.3 (561–655); HWup: 560.6 ± 6.3 (540–596); HW2: 536.2 ± 5.7 (518–572); EL: 175.6 ± 3.3 (162–200); ML: 684.8 ± 10.3 (628–745); PSL: 109.4 ± 2.9 (96–127); SDL: 80.8 ± 2.0 (72–91); MH: 242.8 ± 5.7 (210–269); SPA (in degrees): 60.7° ± 1.6° (51°–68°); PH: 184.3 ± 4.2 (151–205); PL: 179.3 ± 9.6 (90–208); SL: 430.8 ± 4.9 (404–452); HI: 91.2 ± 0.6 (88–94); SI: 130.6 ± 0.8 (127–135).

**Figure 4:**
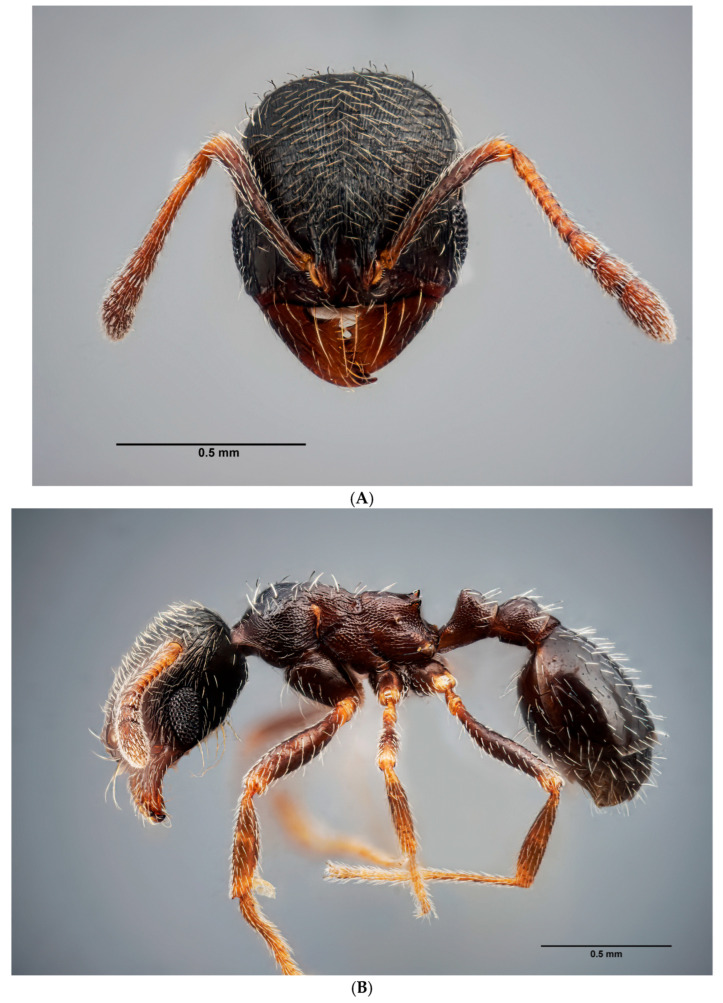

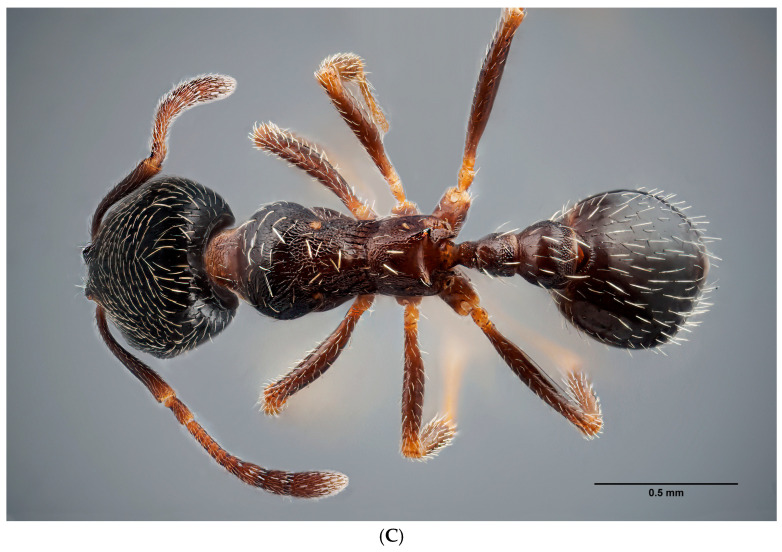
(**A**) Front view of the head of *O. arenarius*. Image by P. Alarcón. (**B**) Side view of the worker of *O. arenarius*. Image by P. Alarcón. (**C**) Dorsal view of the worker of *O. arenarius*. Image by P. Alarcón.

**Coloration**. Head and abdomen dark brown. Thorax and legs brown in color, lighter joints. Antennae dark brown, only apex of the scapes and first segments of funiculus paler (Figure 4A–C).

**Figure 5:**
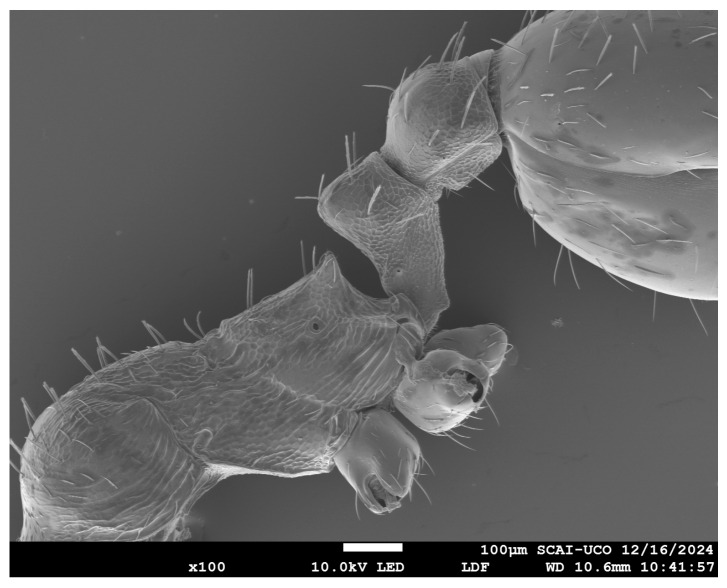
Detail of the mesosoma, lateral view, of *O. arenarius* (SEM microscope). Image by J.L Reyes.

**Head**. Head oval, more long than wide (Figure 4A, Supplementary Figure S1). Antennae with 11 segments; antennal club three-jointed. Mandibles striate, with 7–8 teeth, sometimes the apical tooth massive and long. Compound eyes large, elongated, narrowing downward, reaching the anteroventral margin of the head. Shiny front, with very soft longitudinal striae, sometimes barely perceptible. Gena with either striae and rugae sparser than on frons or smooth, without sculpture, often shinier. General distribution of the setae following the pattern of the genus. Ventral surface of the head with a long psammophore. **Mesosoma**. Dorsal surface of the pronotum shiny, punctuate, with some very short and smooth longitudinal rugae in the posterior part, next to the promesonotal suture (Figure 4B,C). Mesonotum alveolate on the entire surface, lateral surfaces with several transverse striae on the posterior surface, propodeum punctate, with 3–4 distinct longitudinal rugae under the spiracles. Propodeal spines always with a wide base, usually triangular and rising obliquely upward, clearly shorter than in other species of the genus (Figure 5). **Petiolo/Postpetiolo**. Petiole rounded with short peduncle, its anterior face slightly concave, node rounded in profile. Postpetiole in profile regularly rounded (Figure 5). **Gaster**. Gaster bright without micropunctation; with sparse, erect and semierect setae. **Legs**. Legs with short, sparse, appressed setae, inner margin with a row of sparse, long, semierect setae. Tibiae covered with long, appressed-to-semierect setae on the entire surface, inner margins with a row of appressed setae. Very light-colored, yellowish joints.

**Gyne and Male**. Unknown.

**Codes of all captured nests**

B0370: 4w, 28-VIII-2016, Cañada del Pocito, Cartaya, Huelva (37.258800, −7.0668960, 38 m).

B0854: 10w, 19-XI-2022, Laguna del Ojillo, PN Doñana (37.007289, −6.507369°, 25 m).

A4871: 8w, 18-XI-/2017. Laguna de Santa Olaya, PN Doñana (36.981655°, −6.483920°, 8 m).

A5002: 9w, 27-IV-2018, Laguna de Santa Olaya, PN Doñana (36.981655°, −6.483920°, 8 m).

A5003: 8w, 27-IV-2018. Laguna de Santa Olaya, PN Doñana (36.981572°, −6.483313°, 8 m).

**Biological data**

This species has been captured in the Doñana National Park and in the pine forests of Cartaya, always in areas with sandy soil. After rain, the nests form a very characteristic cone, 2–3 cm wide at the base and the same height. Without recent rain, the nest entrance is a simple 2–3 mm hole in the ground. Without workers entering or exiting it, it is impossible to locate, given its small diameter. Some workers were observed transporting seeds individually, without forming trails.

The stabilized dune vegetation of Doñana is dominated by halimium rockrose (*Halimium halimifolium* (L.)), whose light-gray foliage gives rise to the name of this plant community (“Monte Blanco”). Other aromatic plants such as rosemary (*Salvia Rosmarinus* L.), thyme (*Thymus mastichina* L.) and lavender (*Lavandula stoechas* L.) are abundant.

In Doñana, the species *O. saulcyi* is also cited [13,14], although with low frequency.

**Distribution**

**Figure 6:**
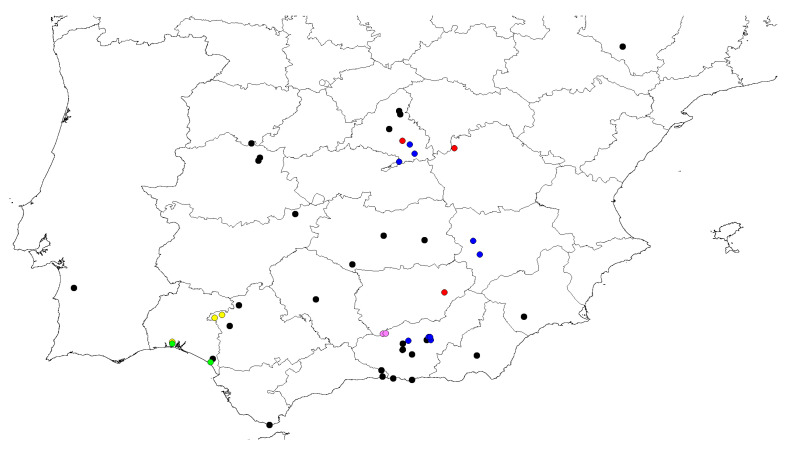
Distribution map of the nests used in the biometric analysis. The green circles correspond to *O. arenaius*, the purple ones to *O. pallipes*, the red ones to *O. magnus* (the blue circles are the currently known locations of this last species) and the yellow ones to *O. saulcy* (the black circles indicate the citations published so far). The distribution map was created using Diva-Gis v.7.5 software (https://diva-gis.org/, accessed on 1 March 2025).

So far, all the captures have been made in the province of Huelva (southern Spain; see Figure 6).

**Differential diagnosis**

Workers are somewhat smaller than *O. saulcyi* and are never bicolored like the latter. They have much smoother, sometimes almost indistinguishable striations on the head and mesosoma, giving them a nearly smooth and shiny appearance. The propodeal spines are shorter and triangular (SPI = 135.6 ± 2.8 (119.1–154.2); SPA = 60.7° ± 1.61° (51.0°–68.0°)). The prodopeum, viewed laterally, has a very straight back.

***Oxyopomyrmex pallens* sp. nov.**

**Etymology**. The epithet “pallens” derives from the Latin {pallens, -entis} and means “pale, colorless”.

**Type locality**. Mures (Jaen). Spain.

**Type material**. Holotype worker (top on the pin): SPAIN|Mures|28-VI-2019|(RLC); two paratype workers: the same data as holotype (MNCN). The rest of the paratypes in the author’s collection.

**Worker description**

**Measurements and ratios** (in microns, *n* = 10): HL: 580.8 ± 10.0 (538–626); HWup: 601.7 ± 9.0 (561–644); HW2: 573.2 ± 9.0 (530–608); EL: 178.7 ± 1.7 (170–185); ML: 715.3 ± 15.7 (639–782); PSL: 144.7 ± 4.4 (123–166); SDL: 86.2 ± 3.8 (63–105); MH: 262.9 ± 7.3 (221–287); SPA (in degrees): 40.5° ± 1.3° (35°-46°); PH: 195.9 ± 3.2 (183–207); PL: 197.5 ± 5.7 (170–212); SL: 421.3 ± 8.5 (377–460); HI: 103.6 ± 0.3 (102–105); SI: 143.0 ± 1.1 (137–150).

**Figure 7:**
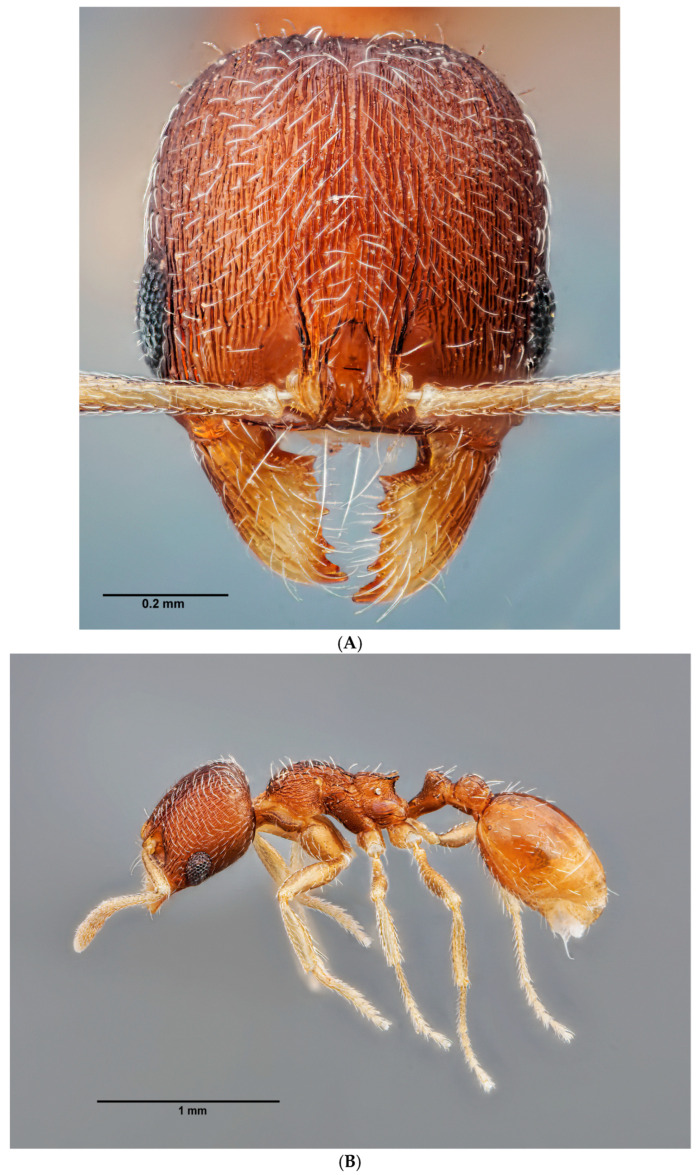

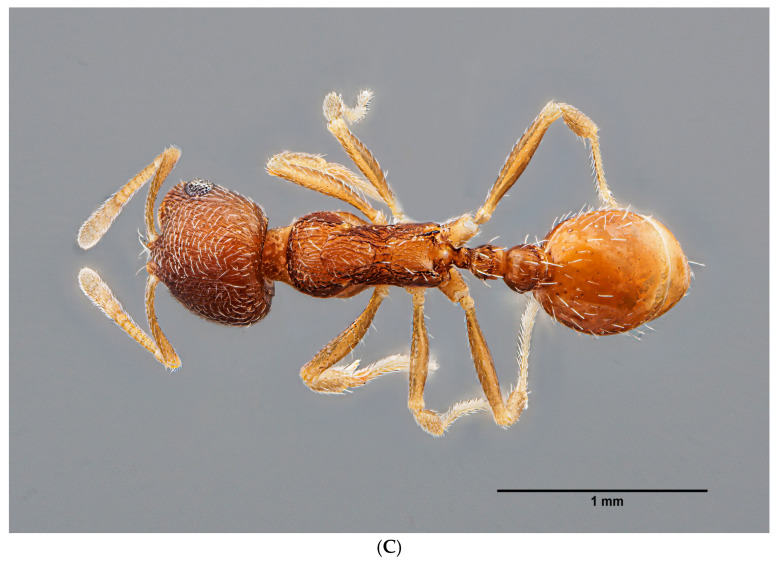
(**A**) Front view of the head of *O. pallens*. Image by P. Alarcón. (**B**) Side view of the worker of *O. pallens*. Image by P. Alarcón. (**C**) Dorsal view of the worker of *O. pallens*. Image by P. Alarcón.

**Coloration**. Head, thorax and abdomen pale brown (Figure 7A–C, Supplementary Figure S2). Antennal scapes pale yellow, apex of the scapes, pedicels and first segments of the funiculus yellowish brown. Mandibles brown to pale brown. Femora pale yellow, tibiae and tarsi yellowish brown. In some specimens, coloration may be darker, approaching black.

**Head**. Head quadrangular, more wide than long, with the lateral surfaces below the eyes straight and gently rounded at the posterior edges (as in *O. magnus*). Frons area smooth and shiny. Typical compound eyes of the genus: longitudinal, strongly narrowed downward, reaching the anteroventral margin. Scape short, 0.7 times as long as the width of the head. Surface of the scape without microsculpture, shiny, covered with short, dense and semierect setae. Clypeus shiny, with some very faint longitudinal rugae. Frontal carinae short, extending to the upper edge of the antennal fossa; antennal fossae smooth without rounded striae, frontal lobes with coarse longitudinal striae, area between striae shiny. Vertex shiny, with the entire surface with coarse longitudinal rugae. Area above eyes rough with thinner longitudinal rugae. Ventral surface of the head with distinctive striations and roughened, gena shiny with longitudinal striations and roughness. Entire head bearing semierect setae, the posterior margin with sparse erect setae directed forward, lateral surfaces of the head with setae directed toward the anterior margin, the frontal area with dense semierect setae placed transversely, directed to the center of the head, the ventral surface of the head with a psammophore. **Mesosoma**. Promesonotum clearly convex in profile. Promesonotal suture distinct. Metanotal groove is well marked. Propodeal spines triangular, rising obliquely upward (Figure 5). Pronotum shiny, rugose with longitudinal striae, lateral surfaces finely rugulose with longitudinal striae. Dorsal surface of pronotum rugose, the area between rugae shiny, lateral surfaces with longitudinal striae and finely rugulose, shiny. Mesonotum rugose and shiny on the dorsum, lateral surfaces rugose with longitudinal striae, the dorsal surface of the propodeum with longitudinal striae or rugose, punctate with longitudinal striae and rugose below spiracles. **Petiolo/postpetiolo**. Petiole node rounded with a short peduncle, its anterior surface concave, clearly rounded node on the dorsal surface when seen in profile. Posterior surface slightly convex. Postpetiole rounded in profile. Base of the petiole and postpetiole on the entire surface punctate, nodes punctate, sparse punctation or micropunctae on the top, shiny. Sides punctate without longitudinal striae, covered with several setae. **Gaster**. Gaster shiny without microreticulation, with erect setae. **Legs**. In some of the workers examined, the legs (and joints) are very light in color, yellowish white.

**Differential diagnosis**

This species is characterized by a head that is wider than it is long (HI > 100). It belongs to the magnus group, which also includes *Oxyopomyrmex magnus*, *O. emeryi* Santschi, 1908, and *O. nitidior* Santschi, 1910 [3]. Within this group, the new species is clearly distinguishable from *O. nitidior* by its light coloration (light brown), as well as by the presence of pronounced striations covering the entire surface of the head, with no smooth areas on the frons or occipital region. This trait is consistent across all examined specimens.

It differs from *O. magnus* primarily in coloration and in the absence of prominent rugae on the mesosoma. Additionally, *O. magnus* is significantly larger. Compared to *O. emeryi*, the new species can be distinguished by the lack of a shiny, finely punctate pronotum; in *O. emeryi*, the entire surface of the pronotum is smooth or micropunctate, particularly on the lateral areas. The maximum size measured so far for ML is 782 microns. For *O. magnus*, the minimum value is 799 µm according to our data (844 µm in [3]). There appears to be a clear size separation between the two species. The size range of *O. nitidior* is between 704 and 894 µm [3].

**Gyne and Male**. Unknown.

**Biological data**. Nothing is known about the biology of this species. The nests were in secondary pastures used for livestock farming. There were scattered holm oaks (*Quercus rotundifolia*).

**Distribution**. Spain: mainland (Figure 6).

**Codes of captured nests**

A5289, 17w, 14-VI-2019, Mures, Jaén (37.415170, –3.847481, 840 m).

A5293, 12w, 14-VI-2019, Mures, Jaén (idem).

A5309, 34w, 28-VI-2019, Mures, Jaén (idem).

A5310, 21w, 10-VII-2021, Gumiel, Granada, (37.418762, –3.805206, 848 m).

**Geographical distribution of continental Spanish species**

In the last work of a compilation of citations on this species [6], after the taxonomic revision [3], it was said to be distributed throughout 17 Spanish provinces, Andorra and Portugal, covering almost the entire peninsula (except the northwest). All collected nests are geographically located in the southern part of Spain. *O. saulcyi* exhibits a broader distribution [6], covering nearly all of Spain, and our data contribute several new locality records. In contrast, for *O. magnus*, this study provides new occurrence data for a species about which very little geographical information is available (see Table 1, Figure 6). *O. magnus* continues to show a tendency to nest at high altitudes, exhibiting a marked orophilous character (590–1411 m). We consistently found the excavated nests of this species in open areas, away from forested zones.

**Key to known species of *Oxyopomyrmex* in continental Spain (workers)**

1. Head always wider than long (HI > 100).......................................................................................................................................................................................................................2

- Head longer than wide (HI < 100)...................................................................................................................................................................................................................................3

2. Center of frons rugulose with longitudinal, uniformly distributed striae. Dark brown or black color ...............................................................................................*O. magnus*

- Softer striae on the forehead. Body color is paler, never black......................................................................................................................................................*O. pallipes* sp. nov.

3. Propodeum with a very straight profile in lateral view. Propodeal spines are triangular in appearance (SPA: 60.7° ± 1.6° (51°–68°)), very short. Dorsal surface of pronotum at least partly punctate with or without striation .........................................................................*O. arenarius* sp. nov.

- Propodeum with convex profile. Propodeal spines longer and thinner (SPA: 44.1° ± 1.07° (37°–53°)). Dorsal surface of pronotum distinctly rugulose ................*O. saulcyi*

## 4. Discussion

Finally, the collection of samples in the south of Spain (Andalusia) has allowed the detection of new taxa for the genus *Oxyopomyrmex*. The differences detected at the biometric and morphological levels allow us to conclude that we are clearly dealing with two species that are new to science. With these two new species, the number of species in the Iberian Peninsula (Spain and Portugal) now equals that in Tunisia (four spp.), reinforcing the role of the northwestern Mediterranean as a center of diversification for this genus. The other center of diversification is in the eastern part of the Mediterranean basin, also according to [3].

Was the discovery of new species in the Iberian Peninsula expected? Yes. The region hosts the highest number of endemic species among Mediterranean countries, with 72 species (24%) recorded [15]. Moreover, many of these endemics are restricted to the southern part of Spain and, potentially, parts of Portugal, underscoring the importance of this area for ongoing taxonomic and biogeographic research.

This genus undoubtedly merits in-depth and detailed study in this geographic area. The same is true for its sister genus, *Goniomma*.

## Figures and Tables

**Table 1:** The code and geographical origin of each of the nests used. “w” represents the number of workers. Altitude is expressed as m.a.s.l. (meters above sea level).

Species	Province	Zone	Code	w	Latitude	Longitude	Altitude
*O. arenarius*	Huelva	Doñana National Park	A4871	7	36.981655	−6.483920	8
*O. arenarius*	Huelva	Cartaya	B0370	4	37.258800	−7.066896	38
*O. pallipes*	Jaén	Mures	A5289	5	37.415170	−3.847481	840
*O. pallipes*	Granada	Gumiel	A5310	5	37.418762	−3.805206	848
*O. saulcyi*	Huelva	Gibraleón	A3322	4	37.290022	−7.067571	50
*O. saulcyi*	Huelva	Gibraleón	A3323	4	37.290022	−7.067571	50
*O. saulcyi*	Sevilla	Villargordo	A4610	5	37.655389	−6.421650	443
*O. saulcyi*	Sevilla	Castillo de las Guardas	A2869	4	37.699146	−6.307548	269
*O. magnus*	Madrid	Rivas-Vaciamadrid	A4076	5	40.357633	−3.553602	590
*O. magnus*	Cuenca	Jabalera	A4421	4	40.244112	−2.761733	803
*O. magnus*	Cuenca	Jabalera	A4433	5	40.244112	−2.761733	803
*O. magnus*	Jaen	Jabalcaballo	A4775	5	38.042502	−2.908860	1411

## Data Availability

All biometric data used in this publication can be made available to authors upon request. Requests would only require an email, explaining their purpose.

## Supplementary Materials

The following supporting information can be downloaded at: https://www.mdpi.com/article/10.3390/insects16060581/s1, Figure S1: Frontal view of a worker’s head under SEM (*O. arenarius*). The very light striation pattern can be more clearly observed. Figure S2: Frontal view of a worker’s head under SEM (*O. pallens*). The striation pattern can be more clearly observed.
