# Supplementary material for: The Ant Genus Oxyopomyrmex Wheeler (Formicidae, Myrmicinae) from the Peninsula Iberica: Two New Species and New Distributional Data
Source: Insects. 2025 May 30;16(6):581. doi: 10.3390/insects16060581 (PMC12193364; doi:10.3390/insects16060581)
Supplement: Supplementary file 1 [file insects-16-00581-s001.zip › insects-3610333-supplementary.pdf]

Supplementary Material. Images.

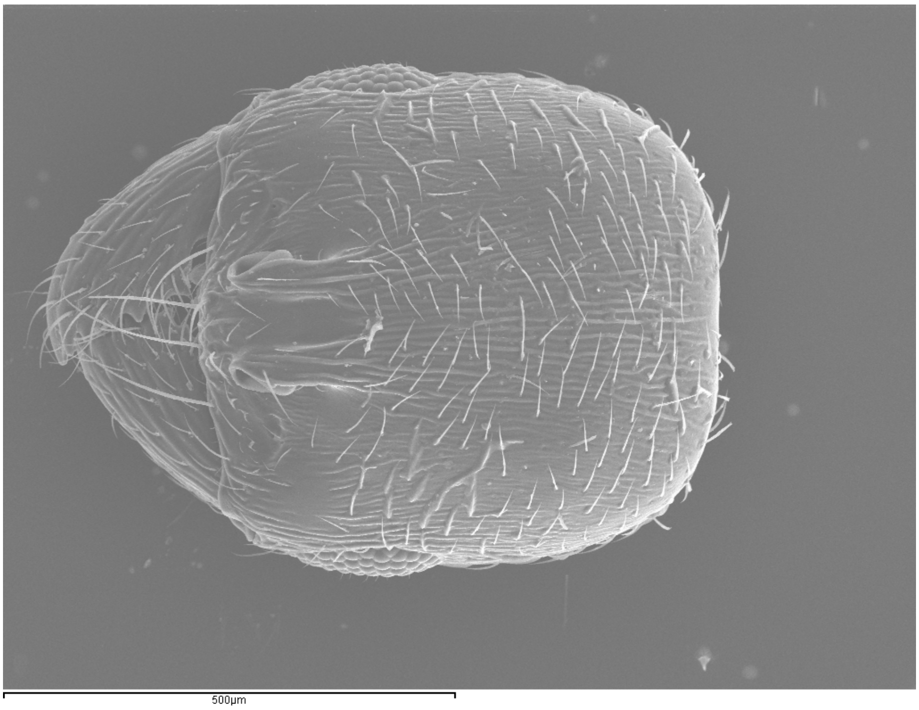

*Figure S1. Frontal view of a worker's head under SEM (**O. arenarius**). The very light striation pattern can be more clearly observed.*

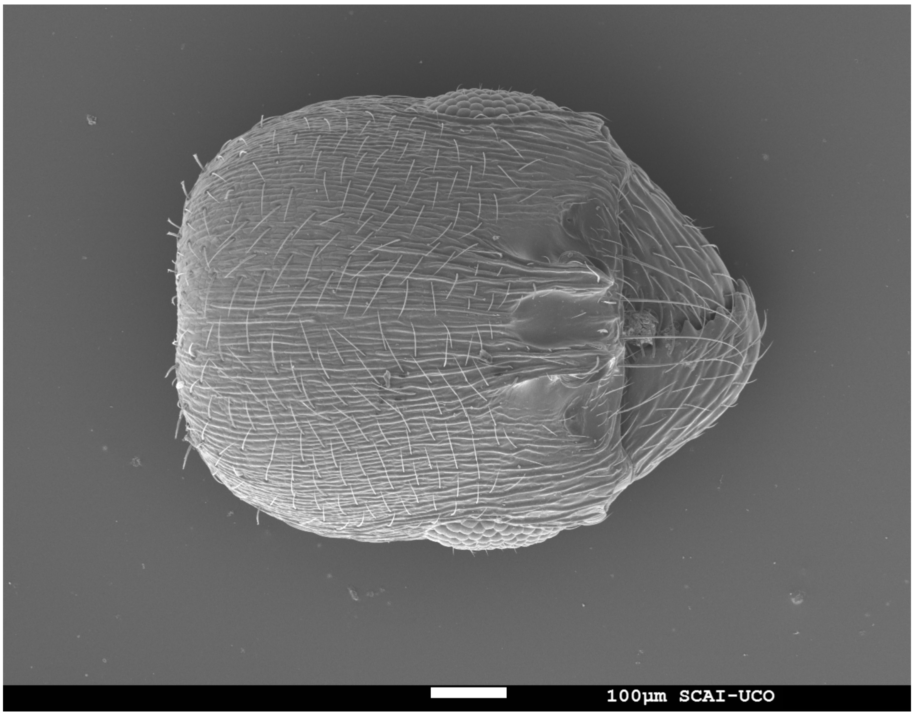

*Figure S2. Frontal view of a worker's head under SEM (**O. pallens)**. The striation pattern can be more clearly observed.*
